# Supplementary material for: Heparin-based hydrogel scaffolding alters the transcriptomic profile and increases the chemoresistance of MDA-MB-231 triple-negative breast cancer cells
Source: Biomater Sci. 2020 Feb 13;8(10):2786–96. doi: 10.1039/c9bm01481k (PMC7497406; doi:10.1039/c9bm01481k)
Supplement: Supplementary file 2 [file BM-008-C9BM01481K-s002.zip › Supplementary File 4/HP-BhydrogelvControl/Pathways/my_analysis.Gsea.1545201079907/HALLMARK_APICAL_JUNCTION.html]

Details for gene set HALLMARK\_APICAL\_JUNCTION[GSEA]

|  || Dataset | expr.class.cls#HYD\_versus\_CONTROL.class.cls#HYD\_versus\_CONTROL\_repos |
| Phenotype | class.cls#HYD\_versus\_CONTROL\_repos |
| Upregulated in class | HYD |
| GeneSet | HALLMARK\_APICAL\_JUNCTION |
| Enrichment Score (ES) | 0.330467 |
| Normalized Enrichment Score (NES) | 1.3189714 |
| Nominal p-value | 0.039800994 |
| FDR q-value | 0.22077866 |
| FWER p-Value | 0.905 |
Table: GSEA Results Summary

  

Fig 1: Enrichment plot: HALLMARK\_APICAL\_JUNCTION      
 Profile of the Running ES Score & Positions of GeneSet Members on the Rank Ordered List

  

| PROBE | DESCRIPTION (from dataset) | GENE SYMBOL | GENE\_TITLE | RANK IN GENE LIST | RANK METRIC SCORE | RUNNING ES | CORE ENRICHMENT || 1 | COL16A1 | na |  |  | 8 | 4.252 | 0.0372 | Yes |
| 2 | MAPK11 | na |  |  | 251 | 2.778 | 0.0491 | Yes |
| 3 | TAOK2 | na |  |  | 355 | 2.559 | 0.0663 | Yes |
| 4 | CLDN14 | na |  |  | 387 | 2.501 | 0.0868 | Yes |
| 5 | SYMPK | na |  |  | 397 | 2.479 | 0.1082 | Yes |
| 6 | IKBKG | na |  |  | 564 | 2.264 | 0.1195 | Yes |
| 7 | WNK4 | na |  |  | 623 | 2.182 | 0.1358 | Yes |
| 8 | COL17A1 | na |  |  | 693 | 2.124 | 0.1510 | Yes |
| 9 | PKD1 | na |  |  | 950 | 1.909 | 0.1544 | Yes |
| 10 | CLDN15 | na |  |  | 974 | 1.891 | 0.1699 | Yes |
| 11 | AMIGO1 | na |  |  | 1035 | 1.851 | 0.1831 | Yes |
| 12 | VAV2 | na |  |  | 1093 | 1.807 | 0.1961 | Yes |
| 13 | SPEG | na |  |  | 1211 | 1.733 | 0.2053 | Yes |
| 14 | EVL | na |  |  | 1426 | 1.618 | 0.2084 | Yes |
| 15 | PLCG1 | na |  |  | 1500 | 1.575 | 0.2185 | Yes |
| 16 | ICAM5 | na |  |  | 1522 | 1.566 | 0.2313 | Yes |
| 17 | INPPL1 | na |  |  | 1587 | 1.538 | 0.2415 | Yes |
| 18 | BAIAP2 | na |  |  | 1657 | 1.508 | 0.2512 | Yes |
| 19 | AKT2 | na |  |  | 1769 | 1.459 | 0.2583 | Yes |
| 20 | NLGN2 | na |  |  | 1811 | 1.437 | 0.2688 | Yes |
| 21 | MVD | na |  |  | 1905 | 1.404 | 0.2764 | Yes |
| 22 | MAP4K2 | na |  |  | 1945 | 1.387 | 0.2866 | Yes |
| 23 | TSPAN4 | na |  |  | 2339 | 1.228 | 0.2768 | Yes |
| 24 | NF2 | na |  |  | 2587 | 1.147 | 0.2740 | Yes |
| 25 | PARD6G | na |  |  | 2744 | 1.105 | 0.2756 | Yes |
| 26 | MAPK13 | na |  |  | 2746 | 1.104 | 0.2853 | Yes |
| 27 | DLG1 | na |  |  | 2806 | 1.083 | 0.2918 | Yes |
| 28 | SHC1 | na |  |  | 3004 | 1.036 | 0.2906 | Yes |
| 29 | MADCAM1 | na |  |  | 3052 | 1.024 | 0.2972 | Yes |
| 30 | LDLRAP1 | na |  |  | 3346 | 0.955 | 0.2903 | Yes |
| 31 | PCDH1 | na |  |  | 3409 | 0.936 | 0.2953 | Yes |
| 32 | IRS1 | na |  |  | 3434 | 0.931 | 0.3023 | Yes |
| 33 | AMH | na |  |  | 3451 | 0.928 | 0.3096 | Yes |
| 34 | CD276 | na |  |  | 3518 | 0.913 | 0.3142 | Yes |
| 35 | ZYX | na |  |  | 3581 | 0.897 | 0.3189 | Yes |
| 36 | VASP | na |  |  | 3608 | 0.892 | 0.3254 | Yes |
| 37 | ADAM23 | na |  |  | 3682 | 0.874 | 0.3293 | Yes |
| 38 | HRAS | na |  |  | 3805 | 0.850 | 0.3305 | Yes |
| 39 | SHROOM2 | na |  |  | 4267 | 0.758 | 0.3130 | No |
| 40 | DHX16 | na |  |  | 4294 | 0.754 | 0.3183 | No |
| 41 | ACTN1 | na |  |  | 4559 | 0.699 | 0.3106 | No |
| 42 | EXOC4 | na |  |  | 4647 | 0.686 | 0.3121 | No |
| 43 | CDH4 | na |  |  | 4989 | 0.628 | 0.2998 | No |
| 44 | ATP1A3 | na |  |  | 5150 | 0.606 | 0.2967 | No |
| 45 | NLGN3 | na |  |  | 5186 | 0.602 | 0.3002 | No |
| 46 | EGFR | na |  |  | 5500 | 0.552 | 0.2887 | No |
| 47 | LAMA3 | na |  |  | 5687 | 0.527 | 0.2835 | No |
| 48 | STX4 | na |  |  | 5734 | 0.521 | 0.2857 | No |
| 49 | BMP1 | na |  |  | 5841 | 0.508 | 0.2847 | No |
| 50 | CDH1 | na |  |  | 5848 | 0.507 | 0.2888 | No |
| 51 | SRC | na |  |  | 6020 | 0.482 | 0.2841 | No |
| 52 | MAPK14 | na |  |  | 6223 | 0.455 | 0.2775 | No |
| 53 | EPB41L2 | na |  |  | 6259 | 0.449 | 0.2797 | No |
| 54 | CLDN18 | na |  |  | 6505 | 0.415 | 0.2705 | No |
| 55 | RHOF | na |  |  | 6593 | 0.404 | 0.2695 | No |
| 56 | THBS3 | na |  |  | 6647 | 0.401 | 0.2703 | No |
| 57 | MYH10 | na |  |  | 6659 | 0.398 | 0.2732 | No |
| 58 | CDK8 | na |  |  | 6702 | 0.393 | 0.2745 | No |
| 59 | GTF2F1 | na |  |  | 6759 | 0.385 | 0.2750 | No |
| 60 | NF1 | na |  |  | 6979 | 0.364 | 0.2667 | No |
| 61 | ICAM2 | na |  |  | 7000 | 0.360 | 0.2688 | No |
| 62 | CD34 | na |  |  | 7160 | 0.341 | 0.2635 | No |
| 63 | ITGA10 | na |  |  | 7287 | 0.328 | 0.2598 | No |
| 64 | CDSN | na |  |  | 7427 | 0.315 | 0.2553 | No |
| 65 | TMEM8B | na |  |  | 7548 | 0.300 | 0.2516 | No |
| 66 | AMIGO2 | na |  |  | 7625 | 0.288 | 0.2502 | No |
| 67 | GNAI1 | na |  |  | 7970 | 0.251 | 0.2343 | No |
| 68 | CDH3 | na |  |  | 8009 | 0.249 | 0.2346 | No |
| 69 | JAM3 | na |  |  | 8155 | 0.239 | 0.2291 | No |
| 70 | MYL9 | na |  |  | 8250 | 0.226 | 0.2261 | No |
| 71 | TRAF1 | na |  |  | 8359 | 0.212 | 0.2223 | No |
| 72 | SDC3 | na |  |  | 8522 | 0.202 | 0.2156 | No |
| 73 | CX3CL1 | na |  |  | 8580 | 0.196 | 0.2144 | No |
| 74 | LAMB3 | na |  |  | 8610 | 0.191 | 0.2145 | No |
| 75 | FBN1 | na |  |  | 8665 | 0.184 | 0.2133 | No |
| 76 | NEGR1 | na |  |  | 8697 | 0.181 | 0.2133 | No |
| 77 | PBX2 | na |  |  | 8846 | 0.159 | 0.2069 | No |
| 78 | TIAL1 | na |  |  | 8854 | 0.159 | 0.2080 | No |
| 79 | TSC1 | na |  |  | 9081 | 0.144 | 0.1974 | No |
| 80 | ICAM1 | na |  |  | 9949 | 0.046 | 0.1523 | No |
| 81 | TNFRSF11B | na |  |  | 10126 | 0.025 | 0.1433 | No |
| 82 | ITGB4 | na |  |  | 10164 | 0.020 | 0.1415 | No |
| 83 | ADRA1B | na |  |  | 10189 | 0.016 | 0.1404 | No |
| 84 | NRTN | na |  |  | 10376 | 0.010 | 0.1307 | No |
| 85 | PTK2 | na |  |  | 10391 | 0.008 | 0.1300 | No |
| 86 | FSCN1 | na |  |  | 10428 | 0.004 | 0.1282 | No |
| 87 | CLDN11 | na |  |  | 10472 | -0.002 | 0.1259 | No |
| 88 | CLDN9 | na |  |  | 10517 | -0.007 | 0.1237 | No |
| 89 | MPP5 | na |  |  | 10543 | -0.009 | 0.1225 | No |
| 90 | ADAM15 | na |  |  | 10681 | -0.027 | 0.1155 | No |
| 91 | CDH15 | na |  |  | 10913 | -0.054 | 0.1039 | No |
| 92 | TUBG1 | na |  |  | 10996 | -0.065 | 0.1001 | No |
| 93 | INSIG1 | na |  |  | 11030 | -0.071 | 0.0990 | No |
| 94 | JUP | na |  |  | 11065 | -0.073 | 0.0979 | No |
| 95 | CD274 | na |  |  | 11286 | -0.087 | 0.0871 | No |
| 96 | CTNND1 | na |  |  | 11317 | -0.091 | 0.0863 | No |
| 97 | CERCAM | na |  |  | 11423 | -0.104 | 0.0817 | No |
| 98 | CRAT | na |  |  | 11449 | -0.107 | 0.0814 | No |
| 99 | LIMA1 | na |  |  | 11620 | -0.128 | 0.0736 | No |
| 100 | LAYN | na |  |  | 11622 | -0.128 | 0.0747 | No |
| 101 | SORBS3 | na |  |  | 11842 | -0.156 | 0.0646 | No |
| 102 | LAMC2 | na |  |  | 11847 | -0.157 | 0.0657 | No |
| 103 | PARVA | na |  |  | 11889 | -0.162 | 0.0650 | No |
| 104 | SLIT2 | na |  |  | 12028 | -0.177 | 0.0593 | No |
| 105 | NEXN | na |  |  | 12128 | -0.190 | 0.0558 | No |
| 106 | GAMT | na |  |  | 12257 | -0.208 | 0.0509 | No |
| 107 | VCL | na |  |  | 12619 | -0.236 | 0.0341 | No |
| 108 | GRB7 | na |  |  | 12640 | -0.239 | 0.0351 | No |
| 109 | PIK3R3 | na |  |  | 13096 | -0.298 | 0.0139 | No |
| 110 | PECAM1 | na |  |  | 13230 | -0.314 | 0.0097 | No |
| 111 | VCAN | na |  |  | 13320 | -0.327 | 0.0079 | No |
| 112 | B4GALT1 | na |  |  | 13415 | -0.340 | 0.0060 | No |
| 113 | GNAI2 | na |  |  | 13525 | -0.357 | 0.0034 | No |
| 114 | WASL | na |  |  | 13860 | -0.412 | -0.0105 | No |
| 115 | RASA1 | na |  |  | 14048 | -0.423 | -0.0165 | No |
| 116 | PPP2R2C | na |  |  | 14388 | -0.475 | -0.0301 | No |
| 117 | CDH11 | na |  |  | 14430 | -0.480 | -0.0280 | No |
| 118 | MDK | na |  |  | 14716 | -0.526 | -0.0384 | No |
| 119 | HADH | na |  |  | 14717 | -0.526 | -0.0337 | No |
| 120 | CNN2 | na |  |  | 14899 | -0.549 | -0.0383 | No |
| 121 | TJP1 | na |  |  | 15122 | -0.585 | -0.0448 | No |
| 122 | CLDN4 | na |  |  | 15213 | -0.598 | -0.0443 | No |
| 123 | AKT3 | na |  |  | 15279 | -0.613 | -0.0423 | No |
| 124 | RRAS | na |  |  | 15432 | -0.638 | -0.0446 | No |
| 125 | RAC2 | na |  |  | 15543 | -0.660 | -0.0445 | No |
| 126 | CLDN7 | na |  |  | 15583 | -0.666 | -0.0407 | No |
| 127 | CTNNA1 | na |  |  | 15914 | -0.726 | -0.0516 | No |
| 128 | MPZL2 | na |  |  | 15948 | -0.732 | -0.0468 | No |
| 129 | YWHAH | na |  |  | 16078 | -0.765 | -0.0468 | No |
| 130 | PFN1 | na |  |  | 16380 | -0.828 | -0.0553 | No |
| 131 | ADAM9 | na |  |  | 16444 | -0.841 | -0.0512 | No |
| 132 | TGFBI | na |  |  | 16797 | -0.927 | -0.0615 | No |
| 133 | MPZL1 | na |  |  | 16841 | -0.939 | -0.0554 | No |
| 134 | MYL12B | na |  |  | 16958 | -0.972 | -0.0529 | No |
| 135 | VCAM1 | na |  |  | 16995 | -0.984 | -0.0461 | No |
| 136 | PTEN | na |  |  | 17107 | -1.016 | -0.0429 | No |
| 137 | ITGA2 | na |  |  | 17270 | -1.074 | -0.0419 | No |
| 138 | CD99 | na |  |  | 17498 | -1.152 | -0.0437 | No |
| 139 | CAP1 | na |  |  | 17535 | -1.168 | -0.0352 | No |
| 140 | ARPC2 | na |  |  | 17586 | -1.191 | -0.0273 | No |
| 141 | PIK3CB | na |  |  | 17903 | -1.335 | -0.0321 | No |
| 142 | CALB2 | na |  |  | 17941 | -1.342 | -0.0222 | No |
| 143 | SIRPA | na |  |  | 18365 | -1.615 | -0.0301 | No |
| 144 | ARHGEF6 | na |  |  | 18548 | -1.744 | -0.0242 | No |
| 145 | RSU1 | na |  |  | 18775 | -1.979 | -0.0186 | No |
| 146 | SKAP2 | na |  |  | 18896 | -2.162 | -0.0057 | No |
| 147 | SGCE | na |  |  | 19030 | -2.435 | 0.0088 | No |
Table: GSEA details [plain text format]

  

Fig 2: HALLMARK\_APICAL\_JUNCTION      
 Blue-Pink O' Gram in the Space of the Analyzed GeneSet

  

Fig 3: HALLMARK\_APICAL\_JUNCTION: Random ES distribution      
 Gene set null distribution of ES for **HALLMARK\_APICAL\_JUNCTION**
